# Intracardiac Mass Reveals Hepatocellular Carcinoma: A Rare Case of Cardiac Metastasis

**DOI:** 10.7759/cureus.97501

**Published:** 2025-11-22

**Authors:** Tarig Abdelrahman, Mohamed Ali, Anika Ahmed, Harini Rangarajan

**Affiliations:** 1 Department of General Medicine, North West Anglia NHS Foundation Trust, Peterborough City Hospital, Peterborough, GBR

**Keywords:** diabetes mellitus, elderly, hepatocellular carcinoma, ischemic heart disease, myocardial infarction

## Abstract

A well-known cause of emergency admission in the elderly is falls, which can be the initial manifestation of severe underlying diseases. This case illustrates an incidental diagnosis of advanced hepatocellular carcinoma (HCC) with vascular invasion after a fall in a middle-aged man.

The patient presented with a mechanical fall at night, with a medical background of severe chronic obstructive pulmonary disease (COPD), type 2 diabetes mellitus, ischemic heart disease, and obesity, at 79 years of age. There was no history of previous chest pain, dizziness, or any neurological problems. On physical assessment, there was bilateral knee swelling with bruising, and a swollen right ankle. Initial studies showed elevated cardiac biomarkers and ECG changes, raising concern for a silent myocardial infarction. A 2D echocardiogram revealed a proliferative right atrial mass and a hepatic lesion, raising suspicion for a tumor. A contrast-enhanced CT scan subsequently confirmed liver cirrhosis and a large hepatic mass extending into the portal vein, inferior vena cava (IVC), and right atrium (RA), consistent with advanced HCC. The patient’s comorbidities and poor performance status prompted the multidisciplinary team to recommend best supportive care only.

This case highlights the importance of a thorough evaluation of elderly patients presenting with falls. It demonstrates how a routine admission can lead to the incidental diagnosis of an advanced malignancy. The case also underscores the need for close attention to unusual symptoms in chronically ill patients, particularly when they may harbor a silent, undiagnosed disease or cancer such as HCC.

## Introduction

Among older people, falls are a common and major source of morbidity, often resulting in hospital admission and triggering formal medical assessment [1]. Although the reasons for falls are frequently related to mechanical instability, neuromuscular weakness, or the side effects of medications, they can sometimes reveal severe underlying pathology [2]. The given case study presents an elderly man who was hospitalized due to a fall at home, which was initially presumed to be mechanical. Nevertheless, subsequent studies identified advanced hepatocellular carcinoma (HCC) with vascular invasion into the portal vein, inferior vena cava (IVC), and right atrium (RA), indicating a serious diagnosis. HCC is the primary type of hepatic cancer and frequently develops in the context of cirrhosis, which can remain silent until significant symptoms occur [3]. Invasion of cardiac structures, although uncommon, is highly aggressive and associated with a poor prognosis. The presented case demonstrates the diagnostic process in geriatric patients with multiple comorbidities and emphasizes the need to maintain a broad differential diagnosis when evaluating non-specific symptoms and non-diagnostic findings during routine medical examinations.

## Case presentation

History and presentation of the patient

Our patient is a 79-year-old gentleman with a complex past medical history comprising severe chronic obstructive pulmonary disease (COPD), type 2 diabetes mellitus, essential hypertension, ischemic heart disease, peripheral vascular disease, moderate aortic stenosis, bronchiectasis, cutaneous squamous cell carcinoma, and obesity (BMI 35). He was admitted to the ED after a mechanical fall in the middle of the night while attempting to use the toilet. He remembered that his legs gave way and he collapsed but denied any loss of consciousness, dizziness, vertigo, syncope, chest pain, or neurological symptoms during the fall. He reported swelling and bruising of both knees along with severe painful swelling of the right ankle. He stated that he had not suffered a head injury, experienced neck discomfort, or had any loss of lucidity after the fall. The next morning, he was unable to bear weight on his legs and had to crawl to bed. The following afternoon, his daughter observed the bruising and swelling and called for an ambulance.

Preliminary examination and investigations

Upon arrival at the hospital, the patient was conscious, alert, and oriented. Cardiovascular examination showed normal heart sounds with a soft ejection systolic murmur in the aortic area. Bilaterally reduced air entry was noted on respiratory examination, consistent with his COPD. There were no signs of neurological deficit. His abdomen was soft and non-tender. Examination of the lower limbs showed swollen, erythematous knees bilaterally, and a bruised, tender right ankle without calf tenderness. A provisional diagnosis of bilateral cellulitis of the knees and a right ankle fracture was made; therefore, he was started on oral flucloxacillin. Imaging ruled out a fracture. However, blood tests demonstrated raised creatine phosphokinase (CPK: 3016 U/L), elevated troponin T (initial 190 ng/L, peaking at 230 ng/L), low platelet count (139 × 10⁹/L), raised CRP (20 mg/L), low magnesium (0.51 mmol/L), and elevated potassium (5.5 mmol/L) (Table 1).

**Table 1:** Laboratory findings on admission with reference ranges.

Test	Patient value	Reference range	Interpretation
Creatine phosphokinase (CPK)	3016 U/L	20-200 U/L	Markedly elevated, suggests muscle injury or infarction
Troponin T (initial)	190 ng/L	<14 ng/L	Elevated, indicates myocardial injury
Troponin T (peak)	230 ng/L	<14 ng/L	Further elevation, confirms ongoing myocardial damage
Platelet count	139 × 10⁹/L	150-400 × 10⁹/L	Mild thrombocytopenia
C-reactive protein (CRP)	20 mg/L	<5 mg/L	Elevated, indicates inflammation/infection
Magnesium	0.51 mmol/L	0.7-1.0 mmol/L	Low, hypomagnesemia
Potassium	5.5 mmol/L	3.5-5.0 mmol/L	Mild hyperkalemia

Sinus rhythm was noted on two consecutive ECGs, with Q waves in V1-V3 and profound T-wave inversions in V4-V6. These changes were not present on earlier ECGs, indicating a silent myocardial infarction. Given his diabetes, he was treated as having an acute coronary syndrome (ACS), and a 2D echocardiogram was ordered.

Incidental discovery of cardiac and hepatic masses

**Figure 1:**
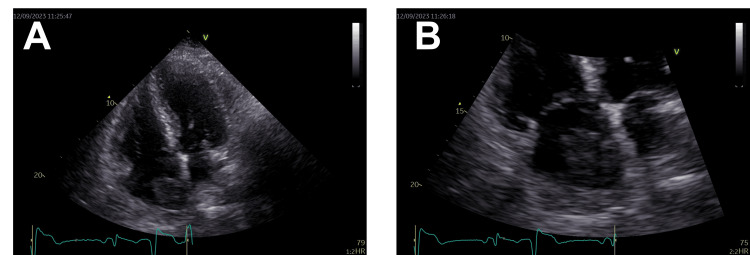
Echocardiographic imaging. A heterogeneous tissue mass (2.7 × 3.5 cm) with frond-like extensions is seen on the roof of the right atrium (RA). A larger vascular hepatic mass (5.7 × 6.2 cm) appears to be in continuity with the right atrial mass.

Surprising results were indicated on the 2D echocardiogram: (1) a heterogeneous tissue mass (2.7 × 3.5 cm) with frond-like extensions on the roof of the RA; (2) a larger vascular hepatic mass (5.7 × 6.2 cm), appearing to be in continuity with the right atrial mass; and (3) moderate aortic stenosis, anterior wall motion abnormalities, an ejection fraction of 40-45%, a dilated right ventricle with preserved systolic function, and enlargement of the ascending aorta at the sinuses and junction (Figure 1). Since the echocardiogram raised suspicion of cardiac involvement secondary to a tumor originating in the liver, an urgent contrast-enhanced CT scan of the abdomen and pelvis was carried out.

Diagnosis CT and MDT diagnosis

**Figure 2:**
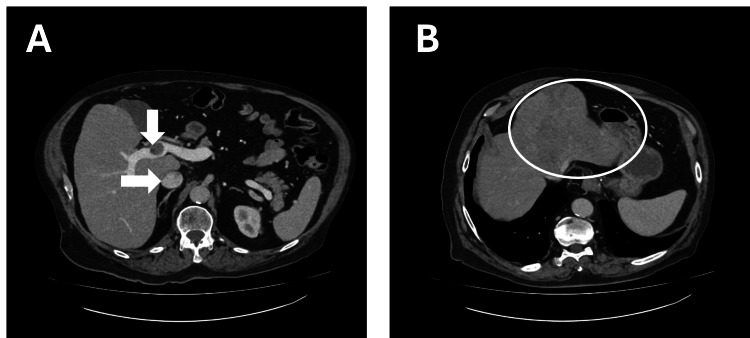
CT imaging demonstrated features of a cirrhotic liver with a large mass in the left lobe, with tumor infiltration into the portal vein, inferior vena cava (IVC), and right atrium. (A) Vascular tumor invasion of the portal vein (left arrow) and the inferior vena cava (downward arrow). (B) Left hepatic mass extending into the portal vein and inferior vena cava.

The CT scan demonstrated features of a cirrhotic liver with a large mass in the left lobe and tumor infiltration into the portal vein, IVC, and RA (Figure 2), findings that were highly suggestive of HCC.

Other primary hepatic neoplasms or metastatic disease were considered as differential diagnoses, but the radiological appearance, together with the patient’s history (alcohol consumption, occupational exposure to carcinogenic agents such as asbestos, and cirrhosis), supported a diagnosis of HCC with vascular invasion.

The case was subsequently discussed in a MDT meeting, and given the patient’s multiple comorbidities, frailty, and poor performance status, it was agreed that best supportive care, rather than curative intervention, would be the most appropriate management.

Follow-up and management plan

The patient attended the outpatient clinic with his daughter. He was in a wheelchair but mentally alert and fully oriented. He reported no symptoms, including no abdominal pain, jaundice, weight loss, or signs of hepatic decompensation. The patient and his family acknowledged and understood the diagnosis.

Macmillan Palliative Services had been contacted for support. Management focused on symptom control, and the patient was initially prescribed low-molecular-weight heparin (LMWH), later switched to rivaroxaban due to the extent of vascular involvement. No surgical intervention or cancer-directed treatment was planned, with ongoing care supervised by the primary physician.

## Discussion

The case represents an unusual but not insignificant manifestation of advanced HCC with tumor growth extending into the RA, diagnosed incidentally following a mechanical fall in an elderly man. HCC, recognized as one of the most common primary liver cancers worldwide, rarely progresses to cardiac involvement, and such findings are usually detected at advanced stages or during autopsies.

Tumor thrombus extension into the IVC and the RA is a rare but well-documented phenomenon of advanced HCC, occurring in approximately 1.4% to 4% of cases based on imaging studies [4,5]. Even higher incidences have been reported in autopsy series, suggesting underdiagnosis during life [6]. In this case, 2D echocardiography revealed a heterogeneous mass with frond-like attachment to the RA, initially raising suspicion for thrombus or a primary cardiac tumor. The continuity of this mass with a vascularized hepatic lesion, subsequently confirmed on contrast-enhanced CT, strongly indicated HCC with direct vascular invasion.

Metastasis or invasion of the heart by HCC carries a very poor prognosis and limited treatment options. Although surgical resection or transarterial chemoembolization (TACE) may offer benefit in highly selected patients [7,8], this patient’s comorbidities and frailty precluded such interventions, making best supportive care the most appropriate approach.

Notably, the patient had no prior symptoms suggestive of liver disease, such as jaundice, abdominal pain, weight loss, bloating, pale stools, or a family history of hepatic malignancy. This is consistent with literature indicating that HCC often remains asymptomatic until it reaches an advanced stage, particularly among individuals with type 2 diabetes, cirrhosis, or non-alcoholic fatty liver disease (NAFLD) [9,10].

In this situation, underlying signs of systemic illness may have been masked by the patient’s diabetes and COPD. The fall, which prompted cardiac and imaging evaluation, ultimately led to the incidental diagnosis. Similar reports describe how falls or non-specific presentations in geriatric patients have revealed underlying malignancies [11,12]. Additionally, it is well known that diabetic patients are prone to silent myocardial infarction [13], which likely explains the elevated troponin and abnormal ECG findings despite the absence of chest pain.

The patient had prolonged occupational exposure to asbestos and cement in the construction sector, which may have indirectly contributed to chronic inflammation and likely played a role in the development of his bronchiectasis and COPD, but is less directly implicated in causing HCC. Nevertheless, some population-based studies have reported an association between environmental and occupational exposures and the pathogenesis of liver malignancies as potential co-factors [14-16].

Prognosis in advanced HCC with vascular invasion is extremely poor, with a median survival of 2 to 4 months without treatment [17]. Given that the patient was considered unsuitable for systemic therapies (e.g., sorafenib or atezolizumab-bevacizumab) due to poor performance status (Eastern Cooperative Oncology Group (ECOG) ≥2) and multiple comorbidities [18,19], these treatments were unlikely to provide meaningful survival benefit. Anticoagulation was initiated to reduce thrombotic risk associated with the tumor mass, although its impact in this context remains unclear.

## Conclusions

The case adds to the small yet growing body of evidence on right atrial infiltration by HCC and demonstrates the importance of thorough assessment in elderly patients with a history of falls and complex medical backgrounds. It highlights the need for caution when interpreting inconspicuous or non-specific results, particularly in diabetic or asymptomatic patients, and illustrates how a seemingly routine clinical presentation can lead to the discovery of an advanced malignancy involving multiple systems.
